# A pilot safety-feasibility dietary trial targeting insulin inhibition in ten patients with advanced cancer

**DOI:** 10.1186/1753-6561-6-S3-P60

**Published:** 2012-06-01

**Authors:** Eugene J Fine, C Segal-Isaacson, Silvia Herzkopf, Joseph Sparano, Maria Romano, Richard Feinman, Nora Tomuta, Amanda Bontempo

**Affiliations:** 1 Nuclear Medicine, Albert Einstein College of Medicine, Bronx, NY, USA; 2 Radiation Oncology, Montefiore Medical Center, Bronx, NY, USA; 3 Medicine, Montefiore Medical Center, Bronx, NY, USA; 4 Cell Biology, Downstate Medical Center, Brooklyn, NY, USA

## Background

Hyperinsulinemia, Diabetes type 2, and obesity have been identified as increased risk factors for a variety of cancers [1]. Conversely, insulin inhibition (INSINH) can plausibly limit cancer growth by demonstrated mechanisms including ketosis [2] and regulation of downstream signaling proteins such as mTOR (inhibition) and AMPK (amplification), already in development as drug targets [3,4]. Increased ^18^F-2-fluoro, 2-deoxyglucose (FDG) uptake on positron emission tomography (PET) scan is characteristic of many aggressive malignancies. We examined safety and feasibility of a four week INSINH diet in patients with advanced PET positive cancers, and compared exit vs. baseline PET scan changes as surrogate measures for tumor response.

## Methods

Eligible patients, referred by faculty or self-referred after locating our trial (e.g. http://www.clinicaltrials.gov/NCT00444054), had failed or refused ≥2 standard chemotherapy courses and demonstrated FDG-positive scans on baseline PET. Exclusions included concurrent chemotherapy, end-organ disease, hypoglycemic medications, difficult compliance, or BMI < 20. A supervised INSINH diet restricting starches and sugars for 28 days, was monitored weekly for macronutrient intake, body weight, serum electrolytes, betahydroxybutyrate concentrations [BHB], [insulin], [IGF 1,2]. An exit four-week PET was obtained for comparison with the baseline scan.

## Results

Ten subjects with diverse cancers completed > 26 days of INSINH without associated unsafe adverse effects. Mean caloric intake decreased (35 ± 6) % vs. predicted requirements despite best efforts to encourage increased food consumption. Weight loss (median 4%, range 0.0-6.1%) was not judged a health risk in any subject. Mild, reversible side effects included constipation (n=2), transient fatigue (n=5), and leg cramps (n=2). Among nine patients with rapid pre-trial progressive disease (PD) five demonstrated post-trial SD or partial remission (SD/ PR) on PET. SD/PR correlated with three-fold higher ketosis compared to those with continued PD (n=4), (p<0.02), but was uncorrelated with reduced calorie intake (p=0.45) or weight loss (p =0.81). Insulin correlated inversely with ketosis (r=0.62, p=0.026), but did not correlate with IFG(1 or 2).

## Conclusions

Preliminary pilot data in ten subjects demonstrated that an INSINH diet is safe and feasible in selected patients with advanced cancer. The extent of ketosis, but neither calorie deficit nor weight loss correlated with SD/PR. The small sample size requires cautious interpretation. Further evaluation is needed to explore the relation of insulin inhibition to calorie restriction, as well as a potential therapeutic role of diet adjunctive to metabolic or cytotoxic therapies.
